# An invasive prey provides long-lasting silver spoon effects for an endangered predator

**DOI:** 10.1098/rspb.2022.0820

**Published:** 2022-06-29

**Authors:** Caroline Poli, Ellen P. Robertson, Julien Martin, Abby N. Powell, Robert J. Fletcher

**Affiliations:** ^1^ Department of Wildlife Ecology and Conservation, University of Florida, Gainesville, FL 32611, USA; ^2^ US Geological Survey, Florida Cooperative Fish & Wildlife Research Unit, PO Box 110430, 110 Newins-Ziegler Hall, University of Florida, Gainesville, FL 32611, USA; ^3^ Department of Natural Resource Ecology and Management, Oklahoma State University, Stillwater, OK 74078, USA; ^4^ US Geological Survey, Wetland and Aquatic Research Center, Gainesville, FL 32653, USA

**Keywords:** natal effects, habitat quality, hydrology, individual condition, residual mass, snail kite

## Abstract

The natal environment can have long-term fitness consequences for individuals, particularly via ‘silver spoon’ or ‘environmental matching’ effects. Invasive species could alter natal effects on native species by changing species interactions, but this potential remains unknown. Using 17 years of data on 2588 individuals across the entire US breeding range of the endangered snail kite (*Rostrhamus sociabilis*), a wetland raptor that feeds entirely on *Pomacea* snails, we tested for silver spoon and environmental matching effects on survival and movement and whether the invasion of a non-native snail may alter outcomes. We found support for silver spoon effects, not environmental matching, on survival that operated through body condition at fledging, explained by hydrology in the natal wetland. When non-native snails were present at the natal site, kites were in better condition, individual condition was less sensitive to hydrology, and kites fledged across a wider range of hydrologic conditions, leading to higher survival that persisted for at least 10 years. Movement between wetlands was driven by the current (adult) environment, and birds born in both invaded and uninvaded wetlands preferred to occupy invaded wetlands post-fledging. These results illustrate that species invasions may profoundly impact the role of natal environments on native species.

## 1. Introduction

Species invasions can influence the environment of native species, altering habitat quality via changes in species interactions and habitat manipulation, leading to changes in fitness, movement and habitat selection of native species [1]. Despite long-standing interest in invasions, the role of invasive species on the natal environment of native species has been largely neglected. Yet, invasive species have the potential to affect the natal environment via negative (e.g. competition) [2] or positive interactions (e.g. facilitation) [3]. Recently, there has been increasing interest in the role of invasive prey for native predators, wherein invasive prey have the potential to provide supplementary resources, and may ultimately increase population growth [3]. For native predators raised in environments with limited native prey, invasive prey may increase natal habitat quality, providing a catalyst for natal environmental effects driven by species invasion.

**Figure 1.**
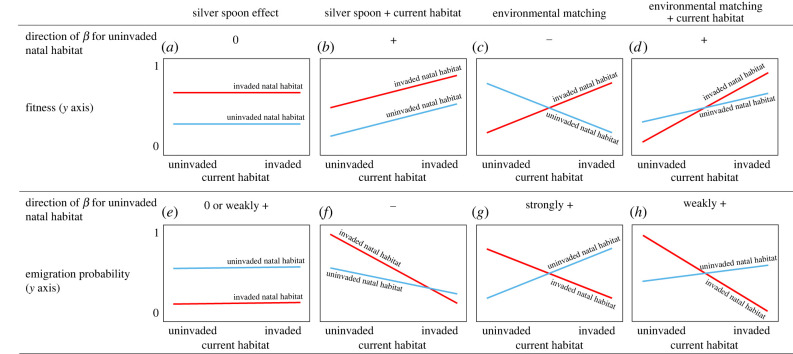
Predictions for the consequences of early life environmental conditions as a function of current (adult) habitat and invasion on fitness (top panels) and emigration (bottom panels). Models for silver spoon effects (*a,e*), silver spoon and current habitat effects (*b,f*), environmental matching (*c,g*) and environmental matching and current habitat (*d,h*). Blue: uninvaded natal habitat; red: invaded natal habitat; *β*: slope parameters. (Online version in colour.)

A variety of hypotheses for the role of the natal environment on organisms may help interpret potential effects of invasive species in the natal environment on native species. One hypothesis is that individuals reared in favourable conditions incur long-lasting benefits, independent of the environment they choose as adults, what has been termed ‘silver spoon effects’ [4] (figure 1*a,b*). Silver spoon effects tend to arise through increases in individual body condition [5], with outcomes of increased survival over time [6] and greater natal dispersal success [7]. According to another hypothesis, natal effects may occur through ‘environmental matching,’ in which the phenotype developed in the natal site allows individuals to perform better when they use natal-like habitat as adults compared to when they do not [8,9] (figure 1*c,d*). These hypotheses may act alone or in concert, yet empirical support for silver spoon effects appears to be stronger than environmental matching [10,11].

The silver spoon and environmental matching hypotheses make contrasting predictions regarding movement that are rarely tested. Habitat selection theory posits that silver spoon effects influence dispersal by allowing individuals from high-quality (invaded) habitat to invest more resources searching for high-quality adult habitat [12]. Silver spoon effects also predict that individuals from low-quality (uninvaded) habitat should have higher movement rates (figure 1*e*). By contrast, environmental matching predicts that individuals will be more likely to disperse to natal-like habitat and achieve higher fitness than in non-natal like habitat (figure 1*g*) [13]. Current (or adult) habitat may override expectations for silver spoon effects and environmental matching such that all individuals will prefer to switch to high-quality habitat, regardless of natal habitat [9] (figure 1*f,h*).

Disperser condition may play a role in silver spoon effects, as individuals in poor condition may have difficulty competing for space or joining an established group within good habitat, and they may also be less selective and therefore more likely to settle in poor habitat [12]. Under silver spoon effects, individuals born in high-quality habitat should have lower movement rates and higher fitness than those born in low-quality habitat. Furthermore, current habitat may widely influence movement [14], such that current habitat effects can occur in combination with natal effects to increase emigration from low-quality areas and decrease emigration from high-quality areas (figure 1). Although preference for natal-like habitat sometimes persists long-term [13], as animals age, current information may become more important for choosing habitat [10]. Given the interplay of age-dependent natal and current effects and their relevance for life-history evolution, it is critical to assess the fitness consequences of the natal environment in the context of movement.

We investigate the role of natural hydrologic conditions and an invasion of non-native prey on natal effects in a native predator, the snail kite (*Rostrhamus sociabilis*). In the US, the snail kite is a wetland-dependent, endangered raptor confined to central and south Florida where it historically fed on a single species of aquatic apple snail (*Pomacea paludosa*). In 2005, a closely related non-native apple snail (*P. maculata*) colonized one wetland within the kite breeding range [15]. By 2009, exotic snails were abundant at nesting sites throughout several wetlands in central Florida [15], and by 2013, they spread across much of the southeastern US [16]. In comparison to native apple snails, *P. maculata* reproduces rapidly, grows larger, is more tolerant to drought, and occurs at higher densities [17,18] (for a comparison of characteristics, see electronic supplementary material, table S1). The tolerance of the exotic snail to drought conditions may be particularly important in the context of natal effects for snail kites, as hydrology plays a key role in nesting habitat suitability and it is actively managed in nearly all wetlands where kites breed [19]. Historically, low water levels led to low availability of native snails [20], but exotic snails could disrupt this relationship. Although *P. maculata* are extremely harmful when they invade freshwater ecosystems [18], they may benefit kites by providing a supplementary prey resource [21]. Recent increases in snail kite nesting activity, juvenile survival, body size, and population size have been linked to the establishment of the exotic snail [15,22], such that invaded habitat is likely higher quality, on average than non-invaded habitat. The natal environment is also known to be important for snail kites: it helps explain dispersal and habitat selection, as well as survival [23,24]. For example, kites are more likely to recruit to and breed in environments that are similar to natal habitat, though reproductive success is highest when birds switch [24]. However, it remains unclear if hydrology and exotic snails in the natal environment have long-term consequences via the silver spoon and/or environmental matching hypotheses.

We compare silver spoon and environmental matching hypotheses to explain how early life conditions can manifest naturally from variation in hydrology and through species invasions using a 17-year dataset across the breeding range of snail kites. We predict that invaded natal habitat will have long-lasting effects consistent with the silver spoon hypothesis because exotic snails have the potential to provide more food and allow kites to fledge in good condition. Individual condition at fledging will therefore be higher in invaded habitat due to abundant prey, as nestling snail kites grow faster in the nest when exotic snails are present [22]. We also predict that hydrologic effects and snail invasion will operate together on individual condition, because water levels can influence prey availability [20], and *P. maculata* is drought-tolerant (electronic supplementary material, table S1), which allows kites to forage in drier conditions. By contrast, if environmental matching is occurring, long-lasting effects will occur when birds use habitat that is similar to natal habitat, regardless of the invasion status of the natal wetland. Prior research revealed that snail kites are more likely to breed in natal-like habitat [24], suggesting environmental matching could be operating; however, these results did not address environmental matching in the context of hydrology and invasive prey.

## 2. Methods

### (a) Study area

**Figure 2.**
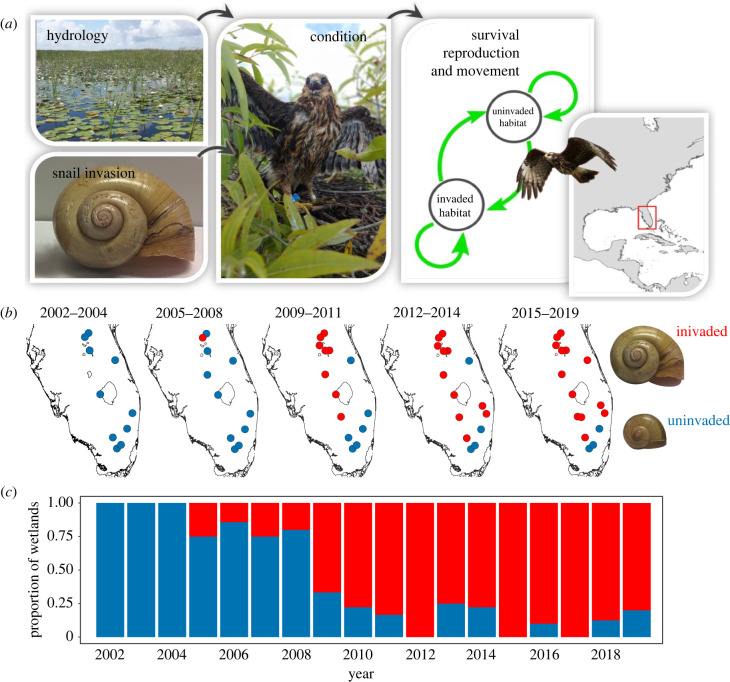
(*a*) The potential pathway for natal effects in snail kites. (*b*) Change in invasion status of 16 wetlands (circles) over time. Shell sizes are proportional to each other. (*c*) The proportion of invaded and uninvaded wetlands each year from 2002–2019. All wetlands were surveyed annually; however, only wetlands that produced fledglings for a given year are shown. Blue: uninvaded, red: invaded. (Online version in colour.)

Our study area includes all known breeding areas for snail kites in the US, which encompasses a network of 16 wetlands in Florida across 35 000 km^2^ (figure 2). Each wetland in each year was classified as uninvaded or invaded using criteria for non-native snail presence [15]. *Pomacea* oviposit conspicuous egg masses on vegetation and human-made structures above the surface of the water, and the eggs of exotic snails are bright pink and easily distinguished from the white eggs of native snails. Invasion status for each wetland and year was determined using exotic snail occurrence data collected systematically during kite surveys, snail shells collected from kite feeding perches and nests, and species inventory surveys.

### (b) Mark–resight and nest monitoring

Surveys for snail kites were conducted throughout wetlands (figure 2) at 2–3 week intervals during peak nesting from 1 March to 30 June 2002–2019. Surveys included counting kites, resighting individually banded kites, finding and monitoring nests, and banding fledglings [19,23]. Upon banding, two feathers were taken from the back and processed via capillary electrophoresis to determine sex. Birds were weighed and measured for wing chord. Snail kites rarely live to 25 years, and drought-induced mortality results in an average lifespan of approximately 8 years [25]. For additional life-history characteristics, see Reichert *et al.* [25].

### (c) Individual and natal site characteristics

We summarized individual condition by calculating the residual mass at fledging [26] for each bird (see electronic supplementary material). To understand hydrology at the natal site, we focused on hydrologic gauges maintained by natural resource agencies, which provide daily measures of the stage (defined as the height of the water surface above an established datum plane) at each wetland. Stage data have been used to successfully interpret nest survival in snail kites [19]. For each breeding wetland, we contacted managers to determine gauges used to guide management activities [22]. We extracted daily stage measures from gauges (https://www.sfwmd.gov/science-data/dbhydro, electronic supplementary material, table S2) at each natal wetland. For each kite that fledged, we calculated mean stage at the natal wetland during the 7 and 14 days prior to fledging. Variation in elevation lead to broad differences in stage measurements among wetlands; therefore, we centred the data for each wetland to the average value across the time period, resulting in a measure of relative wetness/dryness based on stage.

### (d) Analysis

We expected that invasion status of natal habitat would affect survival of kites and movement rates between invaded and uninvaded wetlands independent of the habitat that birds use as adults (current habitat). To estimate survival and movement, we used a multistate mark–recapture model ([27,28]; ‘multistate models’ hereafter). Multistate models simultaneously estimate apparent survival probabilities (*S*; survival hereafter), movement probabilities among states conditional on surviving (*ψ*), and detection probabilities (*p*) [27]. We analysed annual encounter histories for 2,588 snail kites banded near fledging age (figure 2). Kites may move between invaded and uninvaded wetlands; thus, the encounter histories for each bird consisted of two possible states (uninvaded wetland, invaded wetland). Note that many wetlands were invaded during the study, and therefore changed status (figure 2). We allowed survival to vary with current habitat (i.e. stratum: the state of the wetland in the year it was visited, to account for environmental conditions experienced during adulthood). We estimated juvenile and adult survival, but given the annual resolution of the mark–resight data, it was not possible to estimate current effects on juvenile survival or movement (because the encounter for the first year corresponds to natal habitat), so we focus primarily on adults. We tested a categorical covariate for invasion status of natal habitat (invaded/uninvaded), a continuous covariate for individual condition (residual mass), and a continuous covariate for age (in years). To estimate movement rates between invaded and uninvaded wetlands, we allowed *ψ* to vary by current habitat and natal habitat. We tested variation in detection (*p*) three ways: by year, by natal habitat, and a null model, and found the strongest support for year effects based on AICc (*Δ*AICc = 51).

In this modelling context, silver spoon effects on survival would be represented by a model with age class (juvenile, adult) + natal habitat + current habitat or age class + natal habitat × current habitat (i.e. natal habitat influences survival regardless of current habitat). By contrast, environmental matching would be best represented by a model with an effects of age class + natal habitat × current habitat (figure 1), because adult survival is predicted to depend on whether birds match natal with current habitat. Year was included as a nuisance variable to control for annual variability in survival. We also considered six alternative models: age class + natal habitat, age class + current habitat, age class only, year only, age class + year only, and a null model (electronic supplementary material, table S3). We calculated joint survival and movement probabilities (*ϕ*) for each natal and current habitat combination using estimates from the top model. To focus on our hypotheses, we provide estimates based on a median year (2008), but see electronic supplementary material, figure S1 for estimates from all years.

To determine the effects of natal hydrology on individual condition in relation to the invasion status of natal habitat, we used generalized linear mixed models with residual mass as a response variable and mean stage during the 14 and 7 days prior to fledging (hereafter stage), a squared effect of mean stage 14 and 7 days prior to fledging (hereafter stage^2^), and the invasion status of natal habitat (invaded/uninvaded) as explanatory variables (electronic supplementary material, table S4). Linear effects assume that quality either increases or decreases with stage, while quadratic effects allow for the potential that quality is highest at a moderate stage (e.g. low survival at low and high stage), which we expected could occur based on prior research on snail kite nesting [19]. We included random effects of year of fledging and natal wetland to account for non-independence in the data. We tested each response variable six times: with stage as the sole explanatory variable, with stage + stage^2^ as explanatory variables, with an additive effect of the invasion status of natal habitat (invaded/uninvaded) and stage (one model with a linear stage effect and another model with a quadratic stage effect), and with an interactive effect of the invasion status of natal habitat and stage (linear and quadratic).

If silver spoon effects occurred, we expected them to arise from effects of individual condition that may vary with invasion. We tested the top-ranked model from the initial round of selection (age class + natal habitat × current habitat) with an additive effect of condition (electronic supplementary material, table S5). We also allowed for the possibility that survival is highest or lowest for individuals in intermediate condition (using a model with an additive term for condition^2^), and for the possibility that birds in high and low condition are affected in opposite ways by natal habitat (using one model with an interaction between condition × natal habitat, and another model with an interaction between condition^2^ × natal habitat). We considered three alternative models: condition only, age class only, and a null model. We allowed *ψ* to vary by current habitat and *p* to vary by year.

To investigate the longevity of natal effects, we tested the baseline model of natal effects (i.e. natal habitat × current habitat) four ways: with an additive effect of age (in years); with an additive effect of age^2^ (since kite survival over time shows senescence [25]), with an interaction between natal habitat × age (silver spoon effects change over time for individuals from invaded versus uninvaded natal and current habitats), or with an interaction between natal habitat × age^2^ (electronic supplementary material, table S5). We also considered a model of age only, and a null model. We allowed *ψ* to vary by current habitat and *p* to vary by year.

We also explored if natal and current habitat effects could play out on variables other than survival and movement. We considered reproduction (nest success and productivity) in a separate but parallel analysis. A priori, we expected natal environment effects to be greater for survival than reproduction in snail kites because (1) nest success is driven largely by external, current factors (e.g. predation), and (2) productivity has low variability. Overall, there was limited evidence for natal effects on reproductive parameters (see electronic supplementary material).

Throughout, we used model selection to choose among competing hypotheses and ranked models based on Akaike's information criterion adjusted for small sample size [29]. We examined any competing models within 2 AICc of the top model. Median c-hat of a model with no covariates was 2.1. Analyses were conducted in R 3.6 [30] and program MARK [31].

## 3. Results

### (a) Impacts of invasion on survival and movement

**Figure 3.**
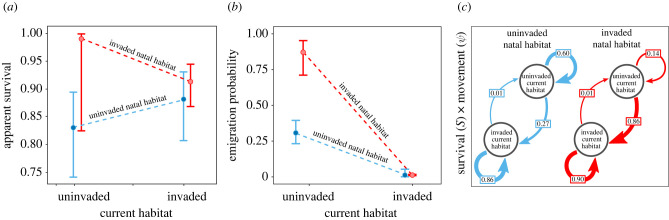
Estimates of (*a*) apparent survival (*S*) and (*b*) emigration probabilities (*ψ*) of 2588 adult snail kites are consistent with silver spoon and current habitat effects due to invaded natal habitat according to the most supported model (electronic supplementary material, table S3). Circle: point estimate, bars: 95% CI; estimates shown are for 2008. (*c*) Joint survival and movement (fidelity and emigration) probabilities *ϕ* (*S* × *ψ*) for birds in invaded and uninvaded wetlands. Arrows: direction of flow, arrow width: strength of flow. (Online version in colour.)

Silver spoon and current habitat effects on apparent survival occurred concurrently based on the most supported model (electronic supplementary material, table S3, figure 1*a,b*), whereas there was no evidence of environmental matching (figure 3*a*). Survival of birds raised in invaded wetlands was higher than those raised in uninvaded wetlands (figure 3*a*; see electronic supplementary material, figure S1 for individual years and electronic supplementary material, table S6 for parameter estimates), and natal habitat appeared to be more important for survival than current habitat, based on comparison of marginal effects (difference in *S* for invaded – uninvaded natal habitat = 8%, invaded – uninvaded current habitat = 4%). Current habitat was most important for individuals raised in invaded wetlands that transitioned to uninvaded current habitat. These birds had the highest survival rate of any group (*S* = 0.98, CI = 0.67–1.0). However, birds rarely moved away from invaded wetlands, so the probability of an individual surviving and moving from an invaded to an uninvaded wetland was extremely low (*ϕ* = 0.01, figure 3*c*). Individuals from uninvaded natal habitat had slightly higher survival when they used invaded current habitat (*S* = 0.79, CI = 0.7–0.86) compared to when they remained in uninvaded habitat (*S* = 0.71, CI = 0.58–0.82). Juvenile survival was lower than adult survival overall, but followed similar relative trends (*S_invaded natal habitat_* = 0.34, CI = 0.25–0.43; *S_uninvaded natal habitat_* = 0.26, CI = 0.17–0.38; electronic supplementary material, figure S2). Annual *p* ranged from 0.3 (CI = 0.17–0.48)–0.76 (CI = 0.71–0.81).

Movements of adults from invaded natal habitat followed expectations for silver spoon and current habitat effects (figure 1*b*). These individuals were highly likely to stay in invaded wetlands (*ψ*^invaded:invaded^ = 0.99, CI = 0.98–0.99) and to move out of uninvaded wetlands (*ψ*^uninvaded:invaded^ = 0.86, CI = 0.7–0.94; figure 3*b*). Movement of individuals raised in uninvaded wetlands was more consistent with current habitat effects. These birds also tended to move away from uninvaded habitat (*ψ*^uninvaded:invaded^ = 0.3, CI = 0.23–0.39) and stay in invaded habitat (*ψ*^invaded:invaded^ = 0.9847, CI = 0.9845–0.9849, figure 3*b*). Counter to findings for survival, current habitat appeared to be more important for emigration than natal habitat, based on marginal effects (difference in emigration estimates for invaded – uninvaded natal habitat = 0.28, invaded – uninvaded current habitat = 0.57). Overall survival (*ϕ*) was strongest for individuals raised in invaded habitat and those that used invaded current habitat (figure 3*c*).

### (b) What drives the silver spoon effect? Hydrology and invasion shape individual condition

**Figure 4.**
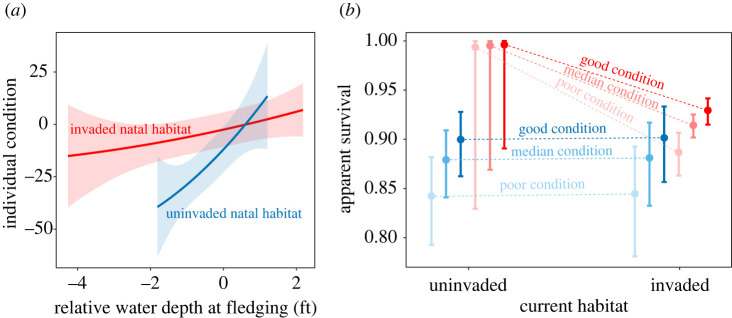
Silver spoon effects on survival are driven by hydrology and occur via individual condition according to the most supported model. (*a*) The effect of natal hydrology (mean relative water depth (stage) in the natal wetland during 7 days prior to fledging) on individual condition (residual mass at fledging; electronic supplementary material, table S4) ± 95% CI. (*b*) The consequences of individual condition for annual apparent survival (electronic supplementary material, table S5) ± 95% CI. Good condition: 97.5^th^ percentile, poor condition: 2.5^th^ percentile, blue: uninvaded natal habitat, red: invaded natal habitat. (Online version in colour.)

Snail kite individual condition at fledging increased in wetter wetland conditions (i.e. increasing wetland stage, a proxy for water depth) and in invaded wetlands, but the magnitude of stage effects dampened with the establishment of exotic snails, based on the most supported model (figure 4*a*; electronic supplementary material, table S4). Variation in condition was also lower in invaded wetlands compared to uninvaded wetlands, particularly because birds exposed to low relative stages fledged in better condition when exotic snails were present (figure 4*a*). Measuring stage over a shorter time frame of 7 days prior to fledging, rather than 14 days, was slightly more important for individual condition based on model selection (electronic supplementary material, table S4).

### (c) Survival increases with individual condition and invasion

Survival was strongly linked to individual condition at fledging, based on the most supported model (electronic supplementary material, table S5). Birds in good condition had higher survival than their counterparts in poor condition, and birds born in invaded natal habitat generally had higher survival that those from uninvaded natal habitat. The advantage of being raised with exotics was so strong that, for birds from uninvaded wetlands, only individuals that fledged in the 97^th^ percentile for condition (i.e. those with residual mass ≥ 43.4) had similar survival probabilities as the poorest birds from invaded habitat (i.e. those in the 5th percentile with residual mass ≤ –68.2). The top condition model also supported a current habitat effect where birds that switched from invaded natal to uninvaded current habitat had high survival.

### (d) Natal effects persist, even as birds age

**Figure 5.**
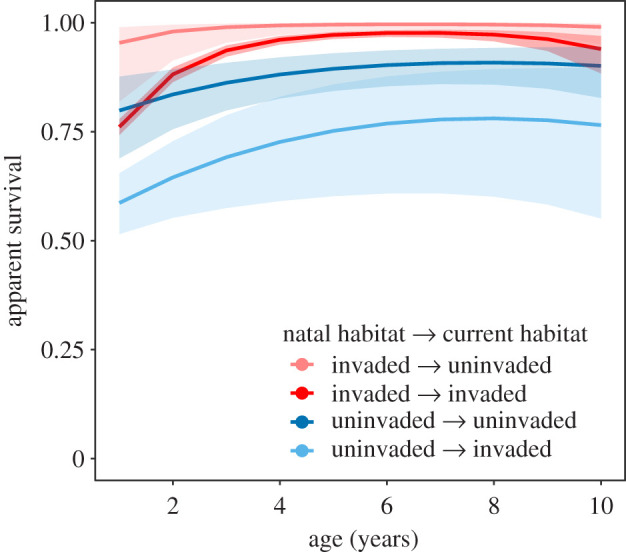
Adult apparent survival is highest for birds born in invaded wetlands, and the effects persist for ≥10 years, according to the most supported model (electronic supplementary material, table S5); estimate ± 95% CI. (Online version in colour.)

Natal habitat effects of the exotic snail on survival were long-lasting: they did not appear to decline with age, and survival was notably higher for kites born in invaded wetlands for individuals up to 10 years old (figure 5), according to the most supported model from model selection (electronic supplementary material, table S5). These differences were greatest at 3–6 years of age where individuals born in an invaded wetland had annual survival rates that were 7.4–30% higher than those born in an uninvaded wetland. Survival rates of birds from invaded wetlands peaked at 5–8 years of age (*S* = 0.98–1), while survival of birds from uninvaded wetlands was highest at 7–9 years of age (*S* = 0.78–0.91).

## 4. Discussion

We found strong evidence for an impact of snail invasion on a silver spoon effect of individual condition at fledging, with long-term consequences for survival. Condition was mediated by two key issues: local hydrology and snail invasion. Individual condition was less sensitive to hydrology when exotic snails were present at the natal site, and early life exposure to exotic snails resulted in higher survival. Survival was particularly high for birds from invaded natal habitat that moved to uninvaded habitat as adults, yet relatively few individuals attempted to switch. Although survival in current habitat depended strongly on whether natal habitat was invaded, movement occurred primarily in response to the invasion status of the current environment. These results underscore that invasive species have the potential to fundamentally alter natal effects in native predators, with long-lasting outcomes [32].

### (a) Species invasion and hypotheses for natal effects on survival and movement

Environmental matching, which may or may not co-occur with silver spoon effects, predicts that individuals that encounter the same conditions in natal and adult habitats are at an advantage because experience gained during early life allows animals to respond appropriately to similar conditions during adulthood [9]. Inconsistent with these assumptions, kites born in uninvaded wetlands had similar survival rates, regardless of whether they switched to invaded habitat or not (i.e. Figure 3*a* is similar to figure 1*a*, not figure 1*c,d*). Environmental matching also supposes that animals from poor-quality habitat are at a disadvantage when colonizing high-quality habitat and incur lower fitness relative to their cohort [9,10]. Kites born in uninvaded habitat were more likely to stay in uninvaded habitat than their counterparts born in invaded habitat; however, once they encountered invaded habitat, they were unlikely to leave. As exotic snails expand into new habitat and uninvaded wetlands become increasingly rare, immigration and fidelity to invaded habitat should increase, regardless of natal origin. For birds born in uninvaded habitat, environmental matching may arise when lack of a competitive advantage in novel habitat decreases the frequency of events where animals in poor condition transition to high quality habitat [33], but prior knowledge for the species does not support this. For example, kites born in lake habitat are more likely to breed in lake habitat, but they incur higher reproductive success when they switch to palustrine wetlands [24], opposite to expectations for reproduction and environmental matching. These results add context to natal habitat preference induction, in which natal habitat shapes future habitat preference [13].

Silver spoon effects can alter expectations for natal effects such that animals born in good-quality habitat have higher fitness, no matter the adult environment [9]. Consistent with silver spoon effects, survival was highest for kites born in invaded habitat. However, it is unexpected that survival of birds that switched from invaded to uninvaded adult habitat was higher than birds that remained in invaded habitat. Several mechanisms may be operating simultaneously. Most importantly, regional effects on survival occur in this system and are somewhat confounded with the progression of snail invasion. Northern wetlands where exotic snails have been established longest (figure 2) tend to have lower adult survival rates, both before and after the invasion [15]. These wetlands tend to be located closer to uplands and urban/suburban areas, which can lead to regional asymmetries in vehicle mortality and predation from mammals or other raptors. Additionally, density dependence may allow birds from invaded, high-density wetlands to successfully recruit to uninvaded wetlands where the relative density of conspecifics tends to be lower. Ultimately, the energetic cost of switching may limit the number of birds that attempt to do so; only 1% of individuals survived and switched from invaded natal habitat to uninvaded current habitat (figure 3*c*). As such switching events were rare, interpreting potential survival benefits should be done cautiously. Continued testing of hypotheses regarding mechanisms that drive habitat choice is essential for understanding the behavioural and evolutionary consequences of natal habitat.

### (b) Invasion mediates hydrologic effects on condition and survival

The silver spoon effect appeared to operate through a chain of interactions in which hydrology at the natal site was related to individual condition at fledging, which in turn was linked to survival. Kites that attained a higher residual mass at fledging had higher survival as adults, and the effect was mediated by the exotic snail. Importantly, kites fledged across a wider range of hydrologic conditions in invaded wetlands and generally had higher condition at fledging across hydrologic conditions than in uninvaded wetlands (figure 4*a*). Interestingly, the observed condition effects could co-occur with increases in morphology due to the exotic snail [22]. Although body condition and morphology are correlated, it would be useful to understand the relative contribution of condition versus morphology *per se* to natal effects. The direct influence of early life body condition and body mass on growth, survival, and reproduction is well known [5,7], but our results add to knowledge of silver spoon effects by highlighting the profound impact that species invasions may have on natal origin and movement of native predators.

In snail kites, condition at fledging may be driven indirectly by water levels, which regulate the activity and abundance of snails and therefore the accessibility of snails to predators [34]. Water levels are also important because kites tend to forage in areas with shallow water and sparse vegetation that can become suboptimal when conditions are too wet or too dry [35]. Additionally, prey availability limits the feeding rate of adults to their offspring, which drives offspring condition [36]. Adjustments in clutch size and brood reduction may also occur in response to local prey availability [37], as is potentially the case for kites that fledge fewer offspring per successful nest when snail density is low [38]. Complex environmental and biological interactions that control the abundance of snails, and snail delivery rates may therefore link hydrology to individual condition and survival. In the long-term, increases in adult survival are hypothesized to occur when animals in good condition recognize high-quality habitat [13], compete for high-quality habitat [12], and invest effort in searching for high-quality habitat [12] compared to animals in poor condition. Where management of kites and wetland habitat is concerned, it is critical to account for hydrology [19], particularly given the strong effects of the exotic snail.

### (c) Invasion confers long-term increases in survival

Our results underscore that silver spoon effects driven by exotic species can have complex, long-term impacts on survival and movement. Specifically, snail invasion at the natal site resulted in long-term increases in adult survival. This relationship played out so that no matter the individual condition at fledging, birds raised with exotic snails gained a relative survival advantage over birds raised with only native snails. Importantly, the benefits to adult survival persisted for nearly 10 years, which is notable given that snail kites senesce at 13 years of age [25]. However, it is important to acknowledge that trade-offs in fitness components are central to life-history evolution [39]. The natal environment may create opportunities for survival at the cost of reproduction [24,40] or lifespan [41] and there may be hidden consequences of invasive species on native species.

### (d) Conservation implications

Our results suggest that in the case of the snail kite, exposure to an invasive species early in life can have long-term benefits by providing supplementary resources at a critical life stage. Invasive prey may have broadly different niches than native prey, causing shifts in predator behaviour that could decrease or amplify potential silver spoon effects. Additionally, silver spoon and current effects observed in this study may not always co-occur. In some cases, differences in natal experience might lead to the presence or absence of one or both effects [9,10]. Given that there is limited information on the mechanisms that lead to positive effects of invasive species, our results provide a valuable example to understand natal effects across an entire breeding range.

These results have implications for management and conservation. Individual condition at fledging provides a potential proxy for long-term survival in kites, which may help in forecasting anticipated future population dynamics and limitations. Additionally, hydrologic effects in wetland systems may play out through variation in water levels within and between sites and years [19,42,43]. A mechanistic understanding of the influence of hydrologic stage at the natal site on fitness can therefore provide a useful tool to guide management of wetland systems. Concerning snail ecology, fluctuations in native snail populations could be attributed to direct competition with the exotic snail [44], but apparent competition might also occur, where increasing kite numbers due to the exotic snail also puts pressure on native snail populations. Across ecological systems, specific knowledge of the underlying drivers of silver spoon effects can provide options for management and conservation of species and habitats despite rapid environmental change.

## Data Availability

The authors confirm that the data (two files) and code (two files) supporting the findings of this study are available within the article and its electronic supplementary material [45].
